# A Dissociation Between Recognition and Hedonic Value in Caloric and Non-caloric Carbonated Soft Drinks

**DOI:** 10.3389/fpsyg.2016.00036

**Published:** 2016-01-29

**Authors:** Franco Delogu, Claire Huddas, Katelyn Steven, Souheila Hachem, Luv Lodhia, Ryan Fernandez, Macee Logerstedt

**Affiliations:** Department of Humanities, Social Sciences, and Communication, Lawrence Technological University, SouthfieldMI, USA

**Keywords:** hedonic valence, carbonated beverages, recognition memory, aspartame, sugar, artificial sweeteners

## Abstract

Consumption of sugar-sweetened beverages (SSBs) is considered to be a contributor to diabetes and the epidemic of obesity in many countries. The popularity of non-caloric carbonated soft drinks as an alternative to SSBs may be a factor in reducing the health risks associated with SSBs consumption. This study focuses on the perceptual discrimination of SSBs from artificially sweetened beverages (ASBs). Fifty-five college students rated 14 commercially available carbonated soft drinks in terms of sweetness and likeability. They were also asked to recognize, if the drinks contained sugar or a non-caloric artificial sweetener. Overall, participants showed poor accuracy in discriminating drinks’ sweeteners, with significantly lower accuracy for SSBs than ASBs. Interestingly, we found a dissociation between sweetener recognition and drink pleasantness. In fact, in spite of a chance-level discrimination accuracy of SSBs, their taste was systematically preferred to the taste of non-caloric beverages. Our findings support the idea that hedonic value of carbonated soft drinks is dissociable from its identification and that the activation of the pleasure system seems not to require explicit recognition of the sweetener contained in the soft drink. We hypothesize that preference for carbonated soft drinks containing sugar over non-caloric alternatives might be modulated by metabolic factors that are independent from conscious and rational consumers’ choices.

## Introduction

The regular consumption of carbonated soft drinks is a very common habit worldwide (Basu et al., 2013). There is evidence that the regular consumption of sugar-sweetened beverages (SSBs) poses a serious public health risk (Malik et al., 2006). For example, people who consume one or more cans of sugar-containing soft drinks daily have a 26% greater risk of developing type 2 diabetes than individuals who rarely consume sugar-containing drinks (Fung et al., 2009). Furthermore, longitudinal studies indicate that subjects who consume an average of one can of a sugar-containing beverage per day have a significantly higher risk of death post-heart attack (De Koning et al., 2012).

There is some agreement that replacing SSBs with non-caloric beverages will reduce the risk of obesity (de Ruyter et al., 2012; Ebbeling et al., 2012; Tate et al., 2012, see also Pereira, 2014 for a review). Artificially-sweetened beverages (ASBs), also referred to as non-nutritive sweetened beverages, have become a popular alternative to SSBs in the soft drink market. In 2014, four of the 10 most consumed carbonated soft drinks in the USA were ASBs, occupying 26.6% of the market share within the top-ten brands (Sicher, 2015). However, some experts are skeptical about the new “calorie-free” drinks, believing that ASBs may still be detrimental to people’s health (Ludwig, 2009). For example, they may increase the risks of obesity (Swithers, 2013), and may have carcinogenic effects (Soffritti et al., 2014). Even if such skepticism does not seem fully supported by data (Renwick and Molinary, 2010), it could have been an influential factor in the substantial slump in sales of diet soft drinks in the USA in the last 5 years (Sicher, 2015).

In humans, the ingestion of sugar produces a sweet taste sensation and a rewarding post-ingestive feedback (Katz and Sadacca, 2011). As non-caloric artificial sweeteners elicit sweet taste without providing calories (Low et al., 2014), it is important to understand whether or not the ingestion of ASBs provide analogous levels of post-ingestive pleasure as compared to sugar. Previous studies support the idea that reward can be greater with sugar than with non-caloric sweeteners. For example, Domingos et al. (2011) have found that rodents prefer sweeteners with nutritional values compared to non-nutritive sweeteners, such as sucralose. The preference for sucrose over artificial sweeteners is associated with striatal dopamine release, resulting in a rewarding effect that can be dissociated from sweet taste (see for example Ren et al., 2010). In humans, there are several studies supporting the evidence that sugar and non-caloric sweeteners activate the brain in different fashions. In particular, it has been found that sugar, but not non-caloric sweeteners, is able to activate the reward pathway (Frank et al., 2008; Smeets et al., 2011). Interestingly, Green and Murphy (2012) found different activations in habitual drinkers and non-habitual drinkers of ASBs. Only habitual consumers showed no difference in the brain’s response to both caloric and non-caloric sweet solutions.

Most of the previous studies that tested perceptual discrimination of sugar from non-caloric sweeteners used solutions that had been prepared to fit specific experimental needs (see Schiffman and Gatlin, 1993 for a review). With such methods, Hettinger et al. (1999) tested how well people could recognize familiar tastes without knowledge of what they were tasting. The results indicated that sucrose was correctly labeled as sugar in 66% of the cases, but was mislabeled as aspartame in about 30% of the trials. Aspartame was labeled as artificial sweetener 41% of the time and confused with sugar 50% of the time (Hettinger et al., 1999). The methodological approach of using solutions specifically prepared for the experiment has the advantage of increasing control, but reduces the applicability of results to the actual habits of soft drink consumers. As an alternative, some studies included commercially available drinks in the list of experimental stimuli. Thai et al. (2011) asked their participants to rate the sweetness and the pleasantness of diet and regular Coca-Cola^®^, as well as of many solutions containing different concentrations of sucrose or aspartame. Their findings indicate that the intensity of sweetness perception was greater in Diet Coke^®^ than in regular Coca-Cola^®^, and that regular Coca-Cola^®^ was rated as more pleasant.

The perception of carbonated soft drink taste in naturalistic settings can be influenced by various factors that are independent from chemical senses and metabolic mechanisms. Previous studies have demonstrated that flavor preference in humans is influenced by expectations (Plassmann et al., 2008), crossmodal factors (Imram, 1999; Spence et al., 2010), and branding (McClure et al., 2004). In order to effectively measure sweetener recognition and taste preference while eliminating the influences of intervening factors, such as brand and color of the drinks, all the non-gustatory information should remain unknown to the participants. Considering that replacing SSBs with ASBs may be a factor in reducing sugar consumption, the objective of this study was to contribute to the understanding of the relationship between sweetener recognition and soft drink pleasantness. We aimed at verifying whether the subjective rates of pleasantness and sweetness of carbonated soft drinks is associated with the ability to recognize whether they are sweetened with sugar or with non-caloric sweeteners. Empirically, we tested participants’ accuracy in distinguishing sugar from artificial sweeteners in a set of commercially available carbonated soft drinks and we measured their subjective rates of pleasantness and sweetness.

## Materials and Methods

### Participants

Fifty-five students from Lawrence Technological University (32 men and 23 women), ranging in age from 18 to 34, participated in the study. Average Body Mass Index (BMI) was 22.9 (*SD* = 3.65). The 13.2% of the sample was underweight, 57.9% normal, 28.5% overweight, and 0.3% moderately obese. Eighteen percentage of the participants declared to not to consume carbonated beverages at all, 52% to drink up to three cans per week, 16% to consume four or five cans per week, 10% 6–10 cans per week, and 4% more than 10. Seventy percentage of participants reported to prefer SSBs while 30% to prefer ASBs. The study was approved by the Lawrence Technological University IRB board. As a requirement, all participants signed an informed consent form. None reported any taste or smell problems.

### Materials

Stimuli consisted of 14 commercially available carbonated soft drinks. Stimuli covered a range of flavor variation: Cola, Orange, Lime, Mountain Dew^®^-like, Dr. Pepper^®^-like, Root Beer, and Vanilla. Two different kinds of beverages were selected for each flavor, one sweetened with sugar (High Fructose Corn Syrup) and the other with one or more low-caloric artificial sweeteners. All of the artificially sweetened drinks contained aspartame, either as the sole sweetener or in combination with other artificial non-caloric sweeteners, like Acesulfame potassium. The beverages were selected among the most popular soft drinks in the United States: six of the 14 stimuli are included in the list of the 10 best-selling soft drinks in the United States (Sicher, 2015). The seven SSBs were Fanta^®^, Coke^®^, Dr. Pepper^®^, 7 Up^®^, Mountain Dew^®^, A&W Root Beer^®^, and Vanilla Coke^®^. The seven ASBs were Fanta zero^®^, Coke Zero^®^, Dr. Pepper Zero^®^, Diet 7 Up^®^, Mountain Dew Zero^®^, Diet A&W Root Beer^®^, and Vanilla Coke Zero^®^.

Whenever available, the beverages were purchased in the 12 oz. aluminum can format. If the can format was unavailable, the beverages were bought in plastic bottles. All beverages were stored at a temperature of 4°C and were brought to room temperature shortly before administration. The decision to use actual carbonated soft drinks instead of experimental solutions has strengths and weaknesses. On the one side, using existing drinks exposes the study to risks of reduced control, because the drinks does not vary only in sweeteners, but also in ingredients, flavors and colors. On the other side, we believe that the risk of reduced control in the design is adequately compensated by its ecological valence. In particular, testing the sample with the same stimuli commonly experienced by the reference population can reduce the distance between laboratory data and daily life experience and enhance the external validity of the results.

### Procedure

Data collection involved testing of multiple participants in collective experimental sessions. A total of four sessions were performed including 11, 17, 15, and 12 participants, respectively. Concerning the influence of satiation, as participants took part in the experiment for the limited reward of course extra-credits or a small amount of money, it was not practical to require them to fast for several hours before testing. In order to limit variability in the level of satiety, we decided to run the tests in the afternoon so that it was likely that the majority of students had had lunch before the experiment.

Before the test, 15 mL individual samples of each beverage were poured into opaque cups, in which a label (varying from A to N) corresponded to the beverage. Stimuli were prepared and labeled in a separate room to prevent participants from seeing the labeled cans or bottles. The experimenters who administered the stimuli did not know which label was associated with which beverage. Before testing, participants filled in a survey with demographic questions and with information about their soft drink consumption habits. The task consisted in the ingestion of 15 mL of each of the stimuli. For each trial, participants closed their eyes and raised their dominant hand to be ready to receive the cup from the experimenter. We preferred to request participants to close their eyes instead of blindfolding them because using blindfolds could cause awkwardness and even anxiety in some participants. The participants-to-experimenter ratio was approximately 2 to 1 and, consequently, it was possible to have a fine visual control over the participants’ observance of the requirement of keeping their eyes closed and over the temporal aspect of the administration, with all participants receiving the stimuli simultaneously. No violation of the eyes-closed requirement was reported in any of the sessions. All participants were required to ingest the stimulus while keeping their eyes closed. After ingestion, researchers took the cups from participants and they were allowed to open their eyes. Participants indicated on a response sheet what sweetener they thought was contained in the beverage, how much they liked it on a scale from 1 to 10, and how sweet was it, also on a scale from 1 to 10. After responding, participants ingested a small amount of non-salty crackers (oyster crackers) and a small sip of purified water to neutralize the aftertaste of the stimulus. The inter stimuli onset interval was 2 min. This procedure was repeated for all of the 14 stimuli. The order of presentation of the beverages was randomized between participants.

### Analysis

Two separated one-sample *t*-tests were conducted on the recognition accuracy data of ASBs and SSBs to determine if the accuracy in the sweetener recognition task was significantly different from chance level. *P*-values were corrected for multiple comparisons. Factorial analyses of variance were performed on the three dependent variables *Recognition* (proportion of correct recognition of the sweetener), *Pleasantness* (rate of personal liking), and *Sweetness* (subjective rate of beverage sweetness). For each of the dependent variables, the independent variables were *Sweetener* (ASBs vs. SSBs) as a within-subjects factor, and *Gender* and *Intake* as a between-subjects factor. *Intake* indicates the subject’s weekly intake of carbonated soft drinks per week and includes two levels: low (up to three cans per week) and high (more than three cans per week). BMI was measured for all participants. Three participants were excluded from the final analysis because their accuracy scores in the recognition task were more than two standard deviations below the mean. Consequently, the final analysis was conducted on a sample of 52 participants.

In order to determine the appropriate sample size and obtain a stopping rule in data collection, we collected a partial sample (*N* = 20) and applied the formula (*Z*-score)^2∗^*SD*^∗^(1 – *SD*)/(margin of error)^2^ to the partial data. The *Z*-score, for a confidence level of 95%, was *Z* = 1.96, standard deviation obtained from the variable *accuracy* was *SD* = 0.172 and the chosen margin of error was *MOE* = 10%. The resulting sample size is *N* ≥ 54.71 = 55 participants.

## Results

**FIGURE 1:**
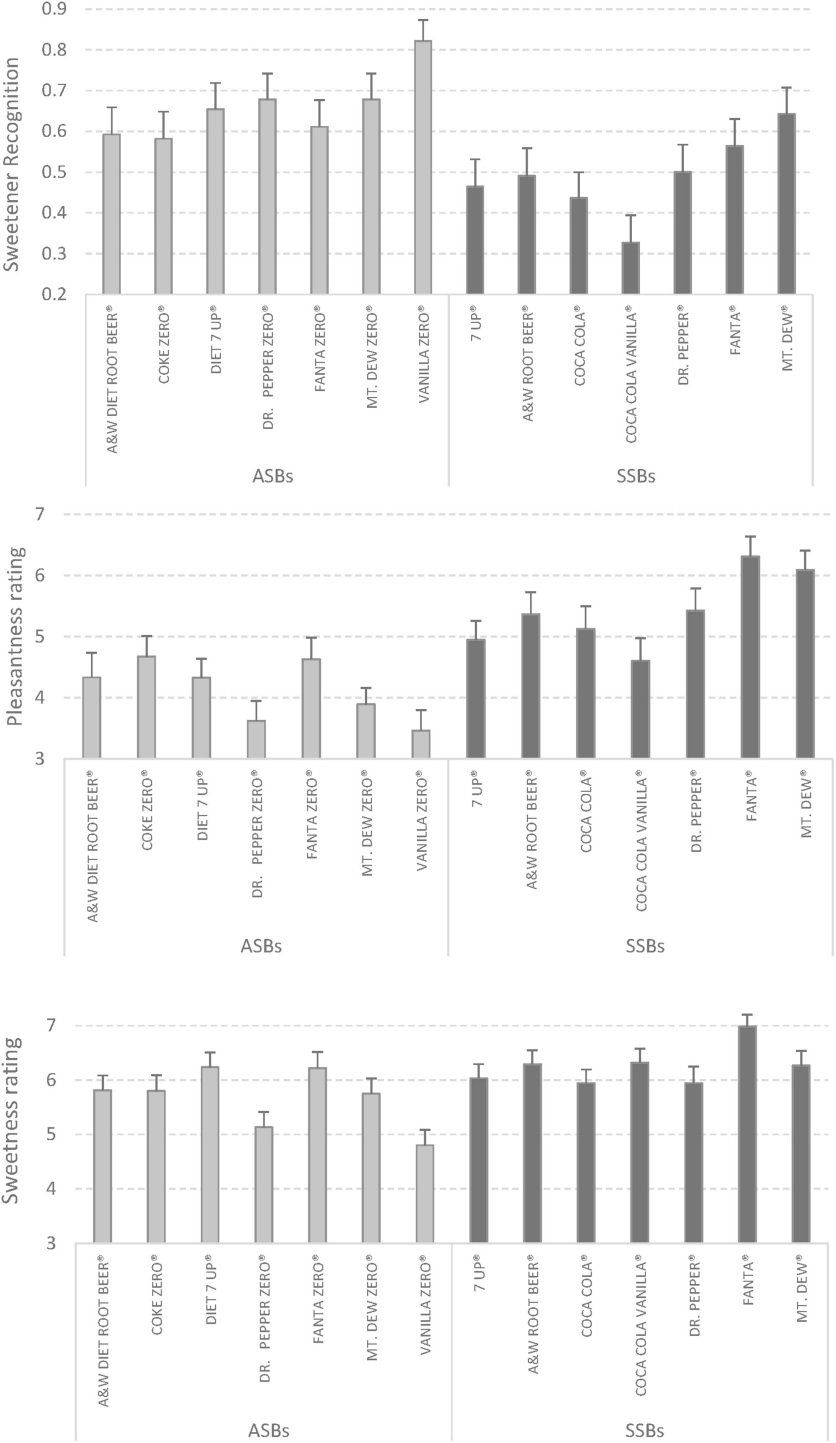
**Sweetener detection accuracy, pleasantness, and sweetness ratings in the 14 beverages.** Error bars represent standard error.

Descriptive results about *sweetness, pleasantness*, and *accuracy* in the detection of the sweetener in all 14 drinks are shown in **Figure 1**.

Concerning *accuracy*, results indicate that recognizing the sweetener contained in the beverages was a difficult task, with an overall 57% of correctly identified stimuli and higher accuracy for beverages containing aspartame (66%) than for beverages containing sugar (52%). Results from one-sample *t*-tests on the recognition accuracy of ASBs and SSBs are reported in **Table 1**. ASBs recognition performance was statistically different from chance level. In contrast, accuracy levels on the SSBs recognition performance does not differ statistically from chance level. This finding suggests that, on average, participants were not able to recognize when beverages were sweetened with sugar while showing a low, but statistically different from chance level recognition of the sweeteners in the ASBs.

**Table 1:** Results of one-sample *t*-test and descriptive statistics for recognition accuracy of artificially sweetened beverages (ASBs) and sugar-sweetened beverages (SSBs).

Outcome	*M*	*SD*	*n*	Comparison value	*t*	*df*	*p*
ASBs recognition accuracy	0.66	0.21	52	0.5	5.52^∗^	51	<0.001
SSBs recognition accuracy	0.52	0.23	52	0.5	0.51	51	0.60

*^∗^*p* < 0.05.*

**FIGURE 2:**
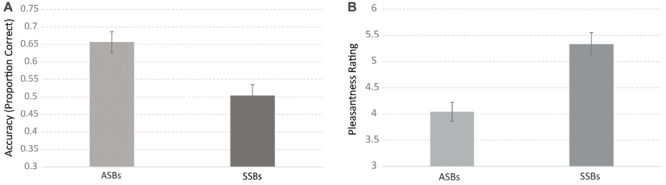
**Average recognition accuracy **(A)** and pleasantness **(B)** as a function of the sweetener.** Error bars represent standard error.

Concerning *Accuracy* (see **Figure 2A**), the main factor Sweetener was significant, *F*(1,42) = 8.67, *p* = 0.005; $\eta_{p}^{2}$ = 0.17, indicating that participants were more accurate in recognizing the sweetener in ASBs than in SSBs. Gender was also significant, *F*(1,42) = 4.85, *p* = 0.03, $\eta_{p}^{2}$ = 0.10, with male participants more accurate than females in the sweetener recognition task. Intake was not significant, *F*(1,42) = 0.18, *p* < 0.67; $\eta_{p}^{2}$ = 0.004, indicating that weekly intake of carbonated soft drinks was not an influential factor in sweetener recognition accuracy. All the 2- and 3-level interactions were not significant.

Concerning *Pleasantness* (see **Figure 2B**), results showed a main effect of Sweetener, *F*(1,43) = 29.60, *p* < 0.00001, $\eta_{p}^{2}$ = 0.40, indicating that SSBs were strongly preferred to ASBs. Gender was not significant, *F*(1,43) = 1.44, *p* < 0.23, $\eta_{p}^{2}$ = 0.03. Intake was also not significant, *F*(1,43) = 0.44, *p* < 0.50, $\eta_{p}^{2}$ = 0.01. All 2- and 3-level interactions were not significant.

Concerning *Sweetness*, SSBs were perceived as significantly sweeter than ASBs, *F*(1,43) = 6.83, *p* = 0.012, η^2^ = 0.14. The main factor Gender was not significant, *F*(1,43) = 2.08, *p* = 0.155, η^2^ = 0.04. Interestingly, the interaction between Sweetener and Gender was significant, *F*(1,43) = 5.59, *p* = 0.022, η^2^ = 0.115. Specifically, a Bonferroni-corrected *post hoc* comparison indicated that male participants perceived SSBs as sweeter than ASBs (*p* = 0.004), while female participants did not (*p* = 1.0). Intake was not significant, *F*(1,43) = 0.75, *p* = 0.389, η^2^ = 0.01.

With regard to the possible role of BMI as an intervening factor, we calculated Person’s *r* correlation values between participants’ BMIs and their accuracy for ASBs (*r* = 0.04), accuracy for SSBs (*r* = 0.16), pleasantness rating for ASBs (*r* = 0.04), pleasantness rating for ASBs (*r* = 0.04), sweetness ratings for ASBs (*r* = 0.04), sweetness ratings for SSBs (*r* = 0.04). None of the correlation values was significant (all *p*-values > 0.05).

In an independent ANOVA analysis, we verified if the preference for either SSBs or ASBs influenced sweetener recognition accuracy, and subjective rates of pleasantness and sweetness. Results indicated that participants, who declared to prefer SSBs had a significantly greater sweetener recognition accuracy, *F*(1,43) = 9.21, *p* = 0.012, η^2^ = 0.16, than the participants who declared to prefer ASBs. *P*-value was corrected for multiples comparisons. The interaction between Sweetener and Preference was not significant. Preference for ASBs or SSBs did not have a significant influence on pleasantness, *F*(1,43) = 1.69, *p* = 0.20, η^2^ = 0.03, or sweetness, *F*(1,43) = 9.21, *p* = 0.90, η^2^

## Discussion

In this study, we asked participants to determine which beverages were sweetened with sugar and which with an artificial sweetener in a series of 14 commercially available carbonated soft drinks. We also asked participants to rate how much they liked the drinks and how sweet they perceived the drinks to be.

Participants showed a low level of discrimination between sugar and artificial sweeteners, with only a 57% overall accuracy. Such a poor performance is consistent with previous evidence of confusion between solutions with similar taste in discrimination tasks (Hettinger et al., 1999). Interestingly, participants were significantly less accurate in detecting sugar than in detecting artificial sweeteners. This result is in contrast with Hettinger’s findings in which sucrose was correctly labeled more times than aspartame. The contrast between results of the two studies may be due to the different kind of stimuli: solutions containing the target substance in isolation in Hettinger’s study versus complex stimuli containing many flavors in our experiment. It is possible that participants could have mistaken SSBs for ASBs as they might have associated loss of carbonation to ASBs. Male participants perceived SSBs as sweeter than female participants. This results supports the existence of gender effects in the perception of the taste intensity (Michon et al., 2009). It is relevant to notice that Michon et al. (2009) found a greater sensitivity to the intensity of sweet taste in female than in male participants.

Concerning the likeability of the drinks, SSBs were strongly preferred to ASBs. This result is consistent with previous evidence of preference of sweeteners with nutrient value compared to non-nutritive sweeteners in humans and other mammals (de Araujo et al., 2008; Domingos et al., 2011). Such preference is likely to be associated with striatal dopamine release and the consequent rewarding effect after consuming sugar, which are absent in non-caloric sweeteners (Frank et al., 2008; Ren et al., 2010; Smeets et al., 2011). Also, participants rated SSBs as sweeter than ASBs, which is consistent with previous findings (Hettinger et al., 1999, but see Thai et al., 2011 for contrasting results).

Previous studies have found that preference for ASBs or SSBs can modulate subjective pleasantness and the activation of the reward system (Green and Murphy, 2012). In contrast with this evidence, we found that preference for caloric versus non-caloric drinks have no influence of on the subjective rating of beverages pleasantness and sweetness. This result is probably associated to the low accuracy of sweetener discrimination. More specifically, the uncertainty about the category to which each drink belonged to, could have counterbalanced the influence of expectations on subjective likeability and perceived sweetness. Additionally, we also found that participants who prefer SSBs were more correct in the sweetener discrimination task. We don’t have a clear account to explain this results.

The most relevant result of this study is the dissociation between the sweetener discrimination accuracy and a systematic preference for drinks containing sugar. The divergence between poor detectability of sugar and the strength of its hedonic value suggests that implicit reward mechanisms are in place when sugar is assumed and that these mechanisms are also active without sweetener recognition. The higher pleasantness rates after ingestion of SSBs can be explained by the activation of the reward system which is selectively activated with sugar but not with non-caloric sweeteners (Smeets et al., 2011). Our findings are consistent with the evidence that levels of liking and the activation of the reward system may be independent from the taste of the beverage (de Araujo et al., 2013). We believe that similar mechanisms may have taken place in our experiment. In fact, it is likely that in a situation of perceptual uncertainty where participants could not clearly categorize the drinks, a significant factor influencing preference was given by the implicit rewarding incentive offered by caloric drinks, but not by the non-caloric ones.

A possible confounding factor is the level of satiety of the participants, which was not controlled in our design. Consequently, it should be noted that we cannot exclude the possible influence of interfering metabolic aspects on our results. Further testing on fasting participants should be conducted to confirm the validity of our findings.

## Conclusion

In this study, we investigated the relationship between sweetener detectability, hedonic value, and sweetness in 14 commercially available carbonated soft drinks. We measured sweetener identification accuracy, subjective pleasantness, and perceived sweetness of the beverages. Results indicate that participants systematically preferred beverages sweetened with sugar and that they rated them as sweeter than beverages sweetened with non-caloric artificial sweeteners. Crucially, however, participants were unable to correctly identify that the beverages were sweetened with sugar. Our results show a dissociation between sweetener recognition and hedonic value which suggest that the activation of the pleasure system seems not to require identification of sugar-sweetened soft drinks. Our results extend to an ecological setting the validity of evidence on animals (Ren et al., 2010) and humans (de Araujo et al., 2013) which showed a dissociation between taste sensation on the one side and subjective liking and the activation of the brain’s reward system on the other side. We conclude that preference for carbonated soft drinks containing sugar over low-caloric alternatives might be activated by metabolic factors that are independent of conscious and rational consumers’ choices. As an excessive consumption of SSBs (Fung et al., 2009; De Koning et al., 2012), can constitute a serious health risk our findings can contribute to increasing public awareness of the danger of the implicit rewards associated with sugar-sweetened soft drinks.

## Author Contributions

FD designed the study over an original idea of CH. All authors performed the experiment. FD interpreted the results and wrote the manuscript. All authors actively reviewed the manuscripts.

## Conflict of Interest Statement

The authors declare that the research was conducted in the absence of any commercial or financial relationships that could be construed as a potential conflict of interest.

## References

[B1] Basu S. McKee M. Galea G. Stuckler D. (2013). Relationship of soft drink consumption to global overweight, obesity, and diabetes: a cross-national analysis of 75 countries. *Am. J. Public Health* 103 2071–2077. 10.2105/AJPH.2012.300974 23488503 PMC3828681

[B2] de Araujo I. E. Lin T. Veldhuizen M. G. Small D. M. (2013). Metabolic regulation of brain response to food cues. *Curr. Biol.* 23 878–883. 10.1016/j.cub.2013.04.001 23643837 PMC3767438

[B3] de Araujo I. E. Oliveira-Maia A. J. Sotnikova T. D. Gainetdinov R. R. Caron M. G. Nicolelis M. A. (2008). Food reward in the absence of taste receptor signaling. *Neuron* 57 930–941. 10.1016/j.neuron.2008.01.032 18367093

[B4] De Koning L. Malik V. S. Kellogg M. D. Rimm E. B. Willett W. C. Hu F. B. (2012). Sweetened beverage consumption, incident coronary heart disease, and biomarkers of risk in men. *Circulation* 125 1735–1741. 10.1161/CIRCULATIONAHA.111.067017 22412070 PMC3368965

[B5] de Ruyter J. C. Olthof M. R. Seidell J. C. Katan M. B. (2012). A trial of sugar-free or sugar-sweetened beverages and body weight in children. *New Engl. J. Med.* 367 1397–1406. 10.1056/NEJMoa1203034 22998340

[B6] Domingos A. I. Vaynshteyn J. Voss H. U. Ren X. Gradinaru V. Zang F. (2011). Leptin regulates the reward value of nutrient. *Nat. Neurosci.* 14 1562–1568. 10.1038/nn.2977 22081158 PMC4238286

[B7] Ebbeling C. B. Feldman H. A. Chomitz V. R. Antonelli T. A. Gortmaker S. L. Osganian S. K. (2012). A randomized trial of sugar-sweetened beverages and adolescent body weight. *New Engl. J. Med.* 367 1407–1416. 10.1056/NEJMoa1203388 22998339 PMC3494993

[B8] Frank G. K. Oberndorfer T. A. Simmons A. N. Paulus M. P. Fudge J. L. Yang T. T. (2008). Sucrose activates human taste pathways differently from artificial sweetener. *Neuroimage* 39 1559–1569. 10.1016/j.neuroimage.2007.10.061 18096409

[B9] Fung T. T. Malik V. Rexrode K. M. Manson J. E. Willett W. C. Hu F. B. (2009). Sweetened beverage consumption and risk of coronary heart disease in women. *Am. J. Clin. Nutr.* 89 1037–1042. 10.3945/ajcn.2008.27140 19211821 PMC2667454

[B10] Green E. Murphy C. (2012). Altered processing of sweet taste in the brain of diet soda drinkers. *Physiol. Behav.* 107 560–567. 10.1016/j.physbeh.2012.05.006 22583859 PMC3465626

[B11] Hettinger T. Gent J. Marks L. Frank M. (1999). A confusion matrix for the study of taste perception. *Percept. Psychophys.* 61 1510–1521. 10.3758/BF03213114 10598466

[B12] Imram N. (1999). The role of visual cues in consumer perception and acceptance of a food product. *Nutr. Food Sci.* 99 224–230. 10.1108/00346659910277650

[B13] Katz D. B. Sadacca B. F. (2011). “Taste,” in *Neurobiology of Sensation and Reward* ed. Gottfried J. A. (Boca Raton, FL: CRC Press). 22593911

[B14] Low Y. Q. Lacy K. Keast R. (2014). The role of sweet taste in satiation and satiety. *Nutrients* 6 3431–3450. 10.3390/nu6093431 25184369 PMC4179169

[B15] Ludwig D. S. (2009). Artificially sweetened beverages: cause for concern. *JAMA* 302 2477–2478. 10.1001/jama.2009.1822 19996404

[B16] Malik V. S. Schulze M. B. Hu F. B. (2006). Intake of sugar-sweetened beverages and weight gain: a systematic review. *Am. J. Clin. Nutr.* 84 274–288. 16895873 10.1093/ajcn/84.1.274 PMC3210834

[B17] McClure S. Li J. Tomlin D. Cypert K. Montague L. Montague P. (2004). Neural correlates of behavioral preference for culturally familiar drinks. *Neuron* 44 379–387. 10.1016/j.neuron.2004.09.019 15473974

[B18] Michon C. O’Sullivan M. G. Delahunty C. M. Kerry J. P. (2009). The investigation of gender-related sensitivity differences in food perception. *J. Sens. Stud.* 24 922–937. 10.1111/j.1745-459X.2009.00245.x

[B19] Pereira M. A. (2014). Sugar-sweetened and artificially-sweetened beverages in relation to obesity risk. *Adv. Nutr.* 5 797–808. 10.3945/an.114.007062 25398745 PMC4224219

[B20] Plassmann H. O’Doherty J. Shiv B. Rangel A. (2008). Marketing actions can modulate neural representations of experienced pleasantness. *Proc. Natl. Acad. Sci. U.S.A.* 105 1050–1054. 10.1073/pnas.0706929105 18195362 PMC2242704

[B21] Ren X. Ferreira J. G. Zhou L. Shammah-Lagnado S. J. Yeckel C. W. de Araujo I. E. (2010). Nutrient selection in the absence of taste receptor signaling. *J. Neurosci.* 30 8012–8023. 10.1523/JNEUROSCI.5749-09.2010 20534849 PMC6632684

[B22] Renwick A. G. Molinary S. V. (2010). Sweet-taste receptors, low-energy sweeteners, glucose absorption and insulin release. *Br. J. Nutr.* 104 1415–1420. 10.1017/S0007114510002540 20619074

[B23] Schiffman S. S. Gatlin C. A. (1993). Sweeteners: state of knowledge review. *Neurosci. Biobehav. Rev.* 17 313–345. 10.1016/S0149-7634(05)80015-6 8272285

[B24] Sicher J. (2015). Special issue: U.S. beverage business results for 2014. *Beverage Digest* 66 1–3.

[B25] Smeets P. A. Weijzen P. de Graaf C. Viergever M. A. (2011). Consumption of caloric and non-caloric versions of a soft drink differentially affects brain activation during tasting. *Neuroimage* 54 1367–1374. 10.1016/j.neuroimage.2010.08.054 20804848

[B26] Soffritti M. Padovani M. Tibaldi E. Falcioni L. Manservisi F. Belpoggi F. (2014). The carcinogenic effects of aspartame: the urgent need for regulatory re-evaluation. *Am. J. Ind. Med.* 57 383–397. 10.1002/ajim.22296 24436139

[B27] Spence C. Levitan C. A. Shankar M. U. Zampini M. (2010). Does food color influence taste and flavor perception in humans? *Chemosens. Percept.* 3 68–84. 10.1007/s12078-010-9067-z

[B28] Swithers S. E. (2013). Artificial sweeteners produce the counterintuitive effect of inducing metabolic derangements. *Trends Endocrinol. Metab.* 24 431–441. 10.1016/j.tem.2013.05.005 23850261 PMC3772345

[B29] Tate D. F. Turner-McGrievy G. Lyons E. Stevens J. Erickson K. Polzien K. (2012). Replacing caloric beverages with water or diet beverages for weight loss in adults: main results of the choose healthy options consciously everyday (CHOICE) randomized clinical trial. *Am. J. Clin. Nutr.* 95 555–563. 10.3945/ajcn.111.026278 22301929 PMC3632875

[B30] Thai P. Tan E. Tan W. Tey T. Kaur H. Say Y. (2011). Sweetness intensity perception and pleasantness ratings of sucrose, aspartame solutions and cola among multi-ethnic Malaysian subjects. *Food Q. Prefer.* 22 281–289.

